# Combined eight-long noncoding RNA signature: a new risk score predicting prognosis in elderly non-small cell lung cancer patients

**DOI:** 10.18632/aging.101752

**Published:** 2019-01-19

**Authors:** Runchen Miao, Cuiyun Ge, Xing Zhang, Yang He, Xiaohua Ma, Xiaohong Xiang, Jingxian Gu, Yunong Fu, Kai Qu, Chang Liu, Qifei Wu, Ting Lin

**Affiliations:** 1 Department of Hepatobiliary Surgery, The First Affiliated Hospital of Xi’an Jiaotong University, Xi’an 710061, Shaanxi, China; 2 Department of Respiratory Medicine, Liaocheng People’s Hospital, Taishan Medical College, Liaocheng 252000, Shandong Province, China; 3 Department of General Surgery, Shaanxi Provincial People's Hospital, The Third Affiliated Hospital, Medical College, Xi'an Jiao Tong University, Xi'an 710068, China; 4 Department of Thoracic Surgery, The First Affiliated Hospital of Xi’an Jiaotong University, Xi’an 710061, Shaanxi, China

**Keywords:** long non-coding RNA, NSCLC, prognosis, the elderly

## Abstract

The elderly are the majority of patients with non-small cell lung cancer (NSCLC). Compared to the overall population's predictive guidance, an effective predictive guidance for elderly patients can better guide patients' postoperative treatment and improve overall survival (OS) and disease-free survival (DFS). Recently, the long non-coding RNAs (lncRNAs) have been found to play an important role in predicting tumor prognosis. To identify potential lncRNAs to predict survival in elderly patients with NSCLC, in the present study, we chose 456 elderly patients with NSCLC and analyzed differentially expressed lncRNAs from four Gene Expression Omnibus (GEO) datasets (GSE30219, GSE31546, GSE37745 and GSE50081). We then constructed an eight-lncRNA formula to predict elderly patients’ prognosis in NSCLC. Furthermore, we validated the prognostic values of the new risk model in two independent datasets, TCGA (n=670) and GSE31210 (n=130). Our data suggested a significant association between risk model and patients’ prognosis. Finally, stratification analysis further revealed the eight-lncRNA signature was an independent factor to predict OS and DFS in stage I elderly patients from both the discovery and validation groups. Functional prediction revealed that 8 lncRNAs have potential effects on tumor immune processes such as lymphocyte activation and TNF production in NSCLC. In summary, our data provides evidence that the eight-lncRNA signature could serve as an independent biomarker to predict prognosis in elderly patients with NSCLC especially in elderly stage I patients.

## Introduction

Non-small cell lung cancer (NSCLC) is one of the most common cause of cancer-related death worldwide [1]. As the population ages, the incidence of lung cancer in the elderly population is increasing. According to the cancer statistics in the past decade, approximately 50% of new lung cancer cases were diagnosed in patients older than 65 [2]. About 81% of lung cancer patients worldwide are over 60 years old, accounting for the majority of lung cancer cases [3]. Moreover, there is evidence that age is an important risk factor for NSCLC patients [4]. If the elderly can be prevented in time and receive the optimal treatment, the incidence of lung cancer, even mortality and recurrence rate will be greatly reduced. Therefore, it is necessary to find more targeted diagnostic and prognostic indicators in elderly patients with lung cancer.

Long noncoding RNAs (lncRNAs) are a group of novel RNAs of more than 200 nucleotides in length. Although they have no significant protein-coding capacity, lncRNAs play important roles in regulating gene expression at epigenetic, transcriptional and post-transcriptional levels [5]. Accumulating evidence suggested that lncRNAs play the potential role as novel biomarkers for prognosis prediction in cancers [6–8]. A growing number of lncRNAs are found to be closely associated with patients’ outcome such as XIST, PVT1 and HOTAIR in lung cancer [9–11]. Moreover, the application of risk score models in tumor prognosis is also increasing. In gastric cancer, the 24-lncRNA signature was found to predict patient outcome [12]. Similarly, only two literatures have also found a lncRNA signature predicting prognosis in NSCLC [13,14]. However, the study of outcome-related lncRNA in elderly patients with NSCLC is still in its infancy and requires long-term efforts.

With the rapid development of the big data era, public databases such as The Gene Expression Omnibus (GEO) and The Cancer Genome Atlas (TCGA) provide great help for the analysis of high throughput data and clinical data. By using proper statistical analysis, researchers identified number of prognostic biomarkers of various malignancies. In lung cancer, Meng Zhou et al. [13] verified the prognostic power of eight-lncRNA signature in three non-overlapping independent NSCLC cohorts were obtained from the GEO database. Ting Lin identified a seven-lncRNA signature associated with overall survival in NSCLC through a comprehensive analysis of TCGA and GEO data [14]. However, the lncRNA biomarkers that can effectively predict the prognosis of elderly patients with NSCLC have not been fully elucidated.

Bearing this in mind, in this study, we analyzed 456 elderly patients with NSCLC from GEO database in order to select optimal lncRNAs for prognostic prediction according to the corresponding risk score. Then TCGA dataset and another GEO dataset were used to validate the screened lncRNAs. Furthermore, combining with the clinical characteristics of patients, we explored the potentiality of eight lncRNAs in different clinical subgroups.

## RESULTS

### Identification of eight lncRNAs for prognosis prediction in the training group

After data normalization and combination, a large group comprised of 682 NSCLC samples was constructed based on four GEO datasets (GSE30219, GSE31546, GSE37745 and GSE50081). Out of them, 456 elderly NSCLC patients (age>=60 years) were selected as a training group. Univariable Cox proportional hazards regression analysis was performed to identify certain prognostic related lncRNAs (log2|fold change| >1 and adjusted *P* < 0.05). A total of 281 lncRNAs were chosen for further analyses. Among them, there were 11 lncRNAs significantly correlated with both OS and DFS (both P < 0.01). After adjusted by gender, pathological subtypes, smoking status and AJCC stage by using multivariable Cox proportional hazards regression analyses, eight lncRNAs were finally identified as independent prognostic biomarkers for elderly NSCLC patients. These eight lncRNAs included *LOC284632*, *LINC00869*, *LINC00703*, *LINC00662*, *LINC00324*, *ITGA9-AS1*, *HOXA11-AS*, *DHRS4-AS1*. The detailed information of the above eight lncRNAs were shown in Table 1.

**Table 1:** Eight lncRNAs significantly associated with prognosis of NSCLC patients in the training group.

Gene name	Ensemble ID	Chr.	Coordinate	Coefficient	Hazard ratio		P value	
LOC284632	ENSG00000230023	1	24,200,240-24,211,690	0.690		1.993		0.085
LINC00869	ENSG00000277147	1	149,607,012-149,679,523	0.272		1.313		0.083
LINC00703	ENSG00000224382	10	4,384,246-4,410,612	0.829		2.291		0.064
LINC00662	ENSG00000261824	19	27,790,493-27,793,940	0.076		1.079		0.473
LINC00324	ENSG00000178977	17	8,220,630-8,224,043	-0.646		0.524		<0.0001
ITGA9-AS1	ENSG00000235257	3	37,753,689-37,861,780	-0.445		0.641		0.174
HOXA11-AS	ENSG00000240990	7	27,185,408-27,189,293	0.020		1.020		0.909
DHRS4-AS1	ENSG00000215256	14	23,938,731-23,955,112	-0.204		0.815		0.106

^a^ Derived from the univariable Cox proportional hazards regression analysis in the training group.

^b^ Derived from the multivariable Cox proportional hazards regression analysis in the training group.

### Construction of a lncRNA-based risk score model in the training group

**Figure 1:**
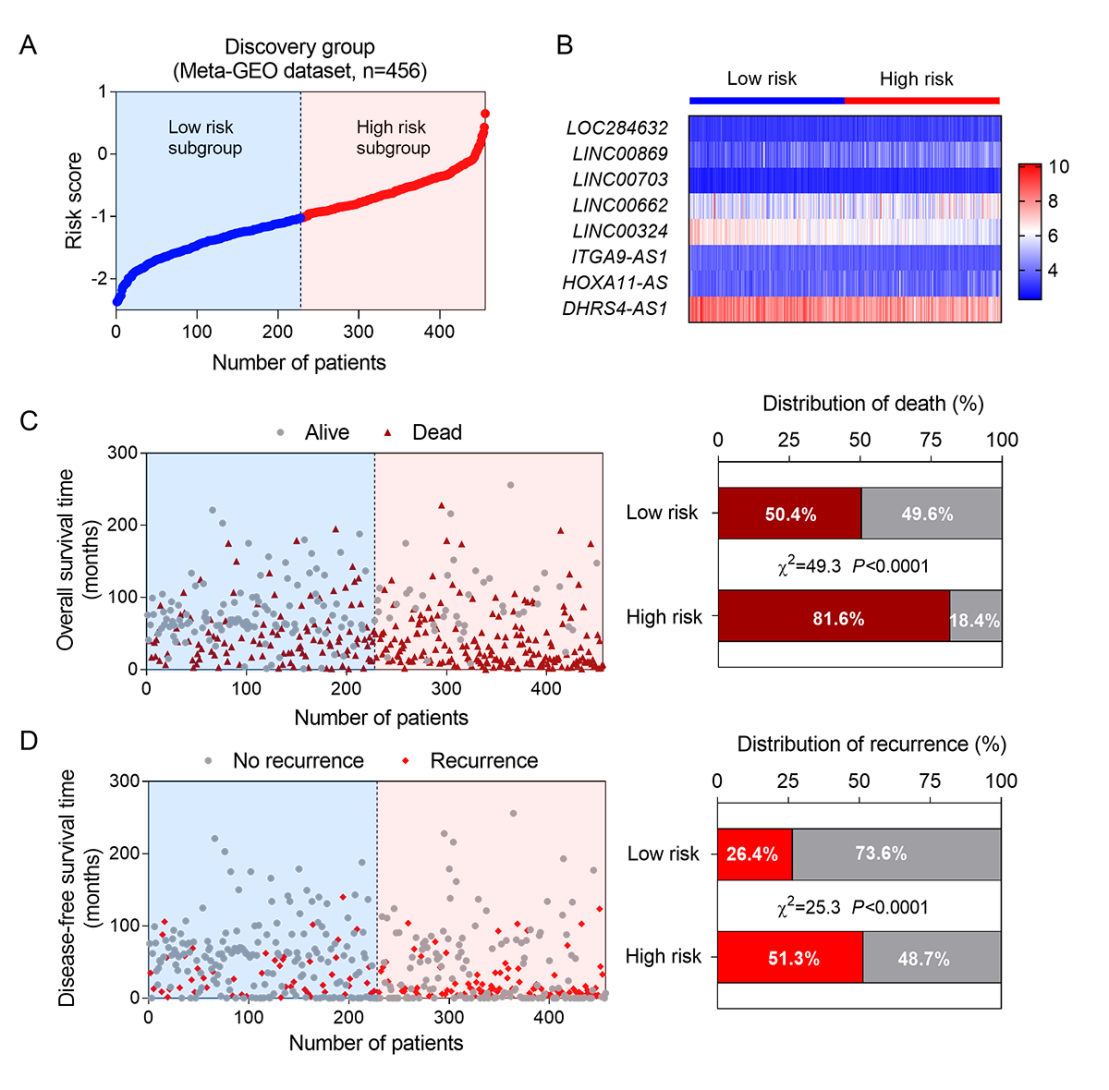
**Construction of a lncRNA-based risk score model in the training group.** (**A**) The eight lncRNA-based risk score distribution; (**B**) Heatmap of the eight-lncRNA expression profiles in the high-risk and low-risk subgroups for the training set.; (**C**) The eight-lncRNA-based risk score distribution for patient survival status (left); the percentage of patient survival status and recurrence in the high-risk and low-risk subgroups of the training set (right); (**D**) The eight-lncRNA-based risk score distribution for patient recurrence (left); the percentage of patient recurrence in the high-risk and low-risk subgroups of the training set (right).

Next, we constructed a prognostic model based on the coefficient of the 8 lncRNAs obtained from multiple Cox regression analysis. The risk-score formula was as followings: risk score = (0.690 × the expression level of *LOC284632*) + (0.272 × the expression level of *LINC00869*) + (0.829 × the expression level of *LINC00703*) + (0.076 × the expression level of *LINC00662*) + (-0.646 × the expression level of *LINC00324*) + (-0.445 × the expression level of *ITGA9-AS1*) + (0.02 × the expression level of *HOXA11-AS*) + (-0.204× the expression level of *DHRS4-AS1*). We calculated the risk scores of 456 patients in training group using above formula. Then the median risk score was used as the cut-off value to divide the training set into two groups, high-risk (n = 228) and low-risk groups (n = 228). The ranked risk scores of patients in the training set was showed as Figure 1A. A heatmap described the expression profiles of these eight lncRNAs in the training group. The samples were ranked according to their risk scores (Figure 1B). Among the 8 lncRNAs, *LINC00324*, *ITGA9-AS1* and *DHRS4-AS1* received a negative coefficient and acted as protective factors. The other 5 lncRNAs with positive coefficients, including *LOC284632, LINC00869, LINC00703, LINC00662* and *HOXA11-AS*, acted as risk factors. In addition, vital and disease status for each patient was plotted, respectively. The proportion of death and recurrence events in different risk groups was also analyzed (Figure 1C-D). In the high-risk group, the patients showed higher mortality and recurrent rate than in the low-risk group.

**Figure 2:**
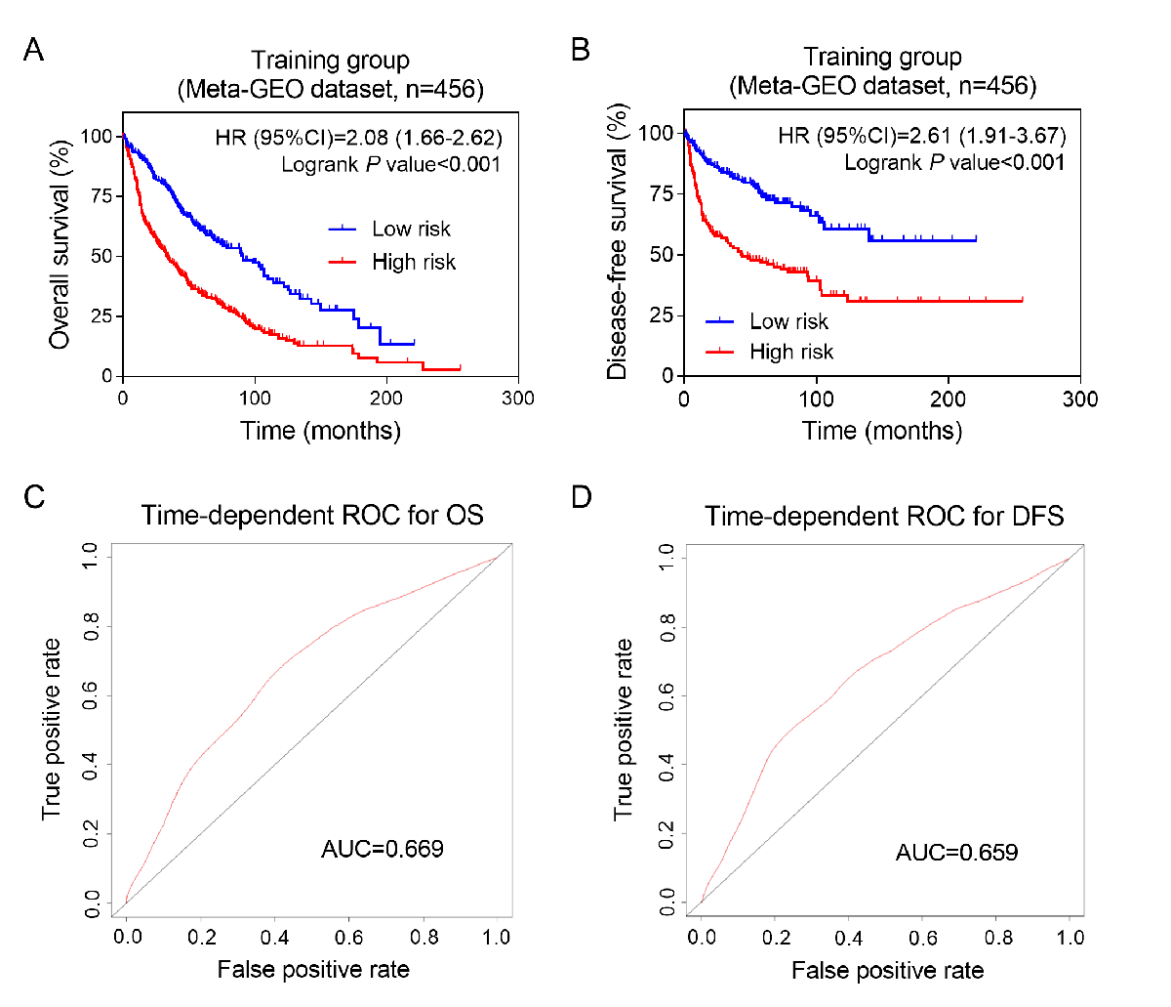
**Prognostic value of eight-lncRNA signature in training group**. Kaplan-Meier analysis of patients’ overall survival (**A**) and disease-free survival (**B**) in the high-risk (n = 228) and low-risk (n = 228) subgroups of the training set; The time-independent ROC analysis of the risk score for prediction the OS (**C**) and DFS (**D**) of the training set. The area under the curve was calculated for ROC curves.

Moreover, Kaplan-Meier analysis was used to evaluate the impact of the above prognosis signature on the survival and recurrence of NSCLC patients in training group. The results showed that the high-risk group had a significantly poorer OS and DFS than that of the low-risk group (Figure 2A-B). We used time-dependent ROC analysis to assess the prognostic significance of eight lncRNAs. The area under the ROC curve (AUC) for the eight-lncRNA signature on OS and DFS was 0.669 and 0.659, respectively, indicating a favorable prognostic value in predict patients' survival (Figure 2C-D).

### The prognostic values of eight lncRNA signature in two independent validation groups

**Figure 3:**
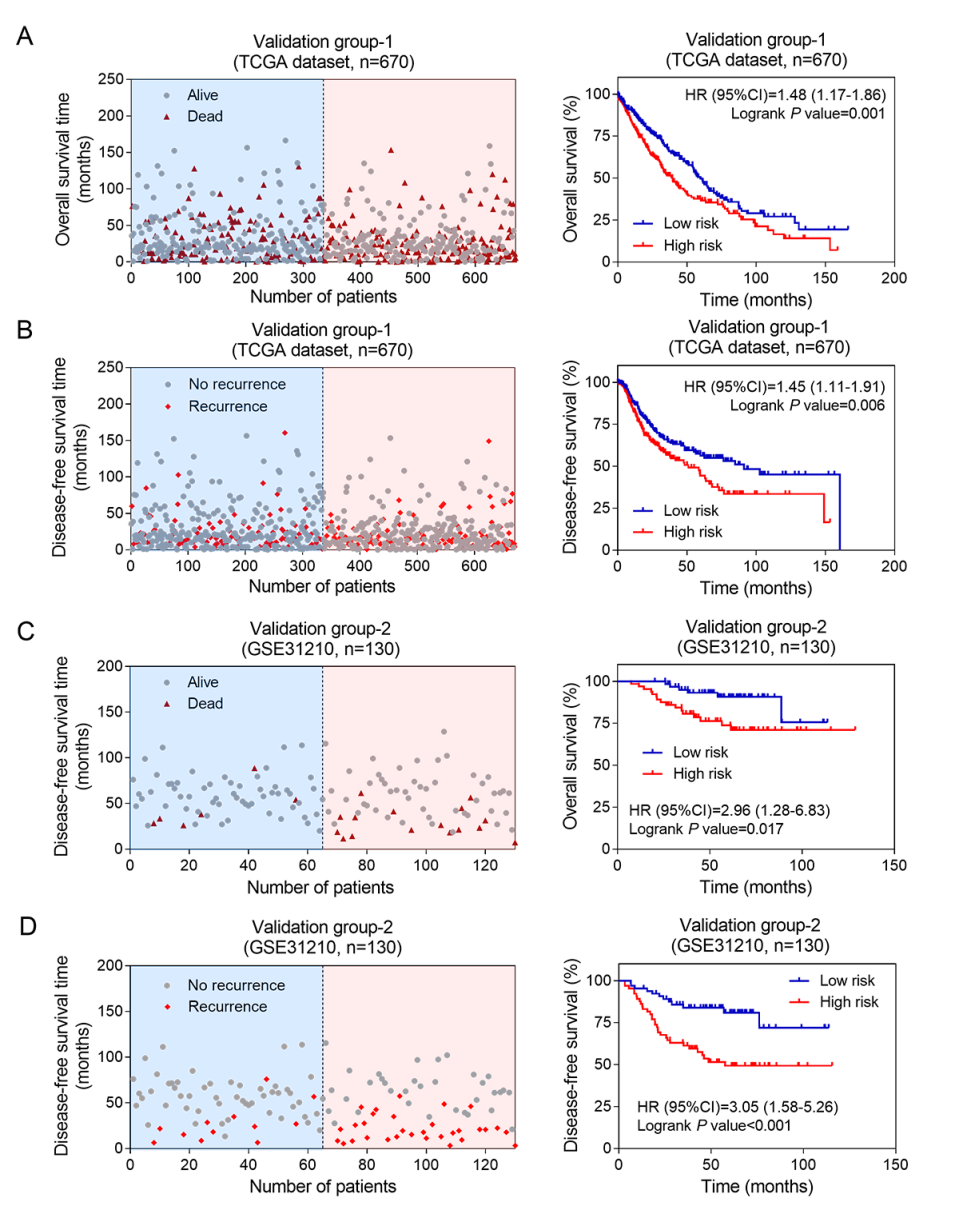
**The prognostic values of eight-lncRNA signature in two independent validation groups.** Kaplan-Meier analysis indicated that patients in the high-risk (n = 335) subgroup exhibited significantly poorer OS (**A**) and DFS (**B**) than the low-risk subgroup (n = 335) in validation group-1; Kaplan-Meier analysis indicated that patients in the high-risk (n = 335) subgroup exhibited significantly poorer OS (**C**) and DFS (**D**) than the low-risk subgroup (n = 335) in validation group-2. The left side shows the distribution of risk scores based on eight-lncRNA in corresponding survival status and recurrence in the two validation groups.

In order to clarify the significance of the above 8 lncRNA in the elderly patients with NSCLC, we used another two independent groups (TCGA dataset and GSE31210 dataset) as validation groups. The corresponding risk score were calculated according to the constructed formula. The elderly NSCLC patients in TCGA (validation group-1, n=670) and GSE31210 (validation group-2, n=130) datasets were divided into high-risk and low-risk groups using dichotomy method, respectively. In validation group-1 and validation group-2, the scatter plots for death and recurrence events were shown in Figure 3. Kaplan-Meier analyses were carried out in validation group-1 (Figure 3A-B). The elderly patients with NSCLC in high-risk group showed worse OS (log-rank test *P* =0.001) and DFS (log-rank test *P* =0.006) than patients in low-risk group. Next, we performed the same analysis on validation group-2 (Figure 3C-D). Consistent with training group results and validation group-1 results, high risk scores on the eight-lncRNA indicated that elderly patients with NSCLC may have a worse OS (log-rank test *P* =0.017) and DFS (log-rank test *P* <0.001). These results demonstrated that the predictive value of the eight-lncRNA signature in elderly patients with NSCLC had a great potential in predicting NSCLC patients’s OS and DFS.

### The eight lncRNAs signature was associated with prognosis in stage I patients

To further investigate the utility of the eight-lncRNA signature, stratification analysis for OS and DFS were performed based on the clinicopathological factors, including gender, smoking status, pathological subtypes and AJCC stage (Table 2 and Table 3). The eight-lncRNA signature had strong predictive power for OS in elder male patients with NSCLC. However, differences between high-risk group and low-risk group for DFS were observed in training group and validation group-2 only. In addition, the eight lncRNAs signature acted as an independent risk factor for patients in both squamous cell carcinoma and adenocarcinoma. This result was only confirmed in training group and validation group-1 because the second validation group did not contain pathological information.

**Table 2:** The association between eight-lncRNA signature and OS of NSCLC patients in discovery and validating groups.

Variable	Discovery Group			Validation Group-1				Validation Group-2			
	Number (High/Low)	HR (95%CI)	*P* value		Number (High/Low)	HR (95%CI)	*P* value		Number (High/Low)	HR (95%CI)	*P* value
**Total**	**228/228**	**2.08 (1.66-2.62)**	**<0.001**		**335/335**	**1.48 (1.17-1.86)**	**0.001**		**65/65**	**2.96 (1.28-6.83)**	**0.017**
Gender											
Male	171/142	1.89(1.46-2.45)	<0.001		209/212	1.46 (1.10-1.94)	0.008		35/25	5.68 (1.41-11.47)	0.010
Female	57/86	2.34 (1.53-3.96)	<0.001		126/123	1.55 (1.03-2.34)	0.036		30/40	1.23 (0.31-4.96)	0.772
Smoking history											
Never smoker	1/16	4.60 (0.49-1458)	0.117		22/29	1.74(0.72-4.17)	0.189		24/42	1.75(0.43-7.65)	0.420
Ever smoker	10/61	0.96 (0.34-2.70)	0.934		217/237	1.382(1.03-1.85)	0.029		41/22	3.889(0.98-8.38)	0.055
Current smoker	8/40	1.11 (0.45-3.10)	0.786		84/59	1.52(0.97-2.36)	0.060		0/0	NA	NA
Histology											
Squamous Carcinoma	141/95	2.04 (1.30-2.86)	0.001		219/196	1.27 (0.96-1.70)	0.099		0/0	NA	NA
Adenocarcinoma	68/152	2.11 (1.60-3.54)	<0.001		116/139	1.97 (1.32-2.95)	<0.001		0/0	NA	NA
AJCC stage											
**Stage I**	**144/166**	**2.12 (1.63-2.88)**	**< 0.001**		**180/174**	**1.68 (1.19-2.38)**	**0.003**		**41/56**	**4.39 (1.32-13.25)**	**0.015**
Stage II	39/50	1.49 (0.88-2.57)	0.138		97/86	1.44 (0.91-2.28)	0.118		24/9	1.29 (0.37-4.37)	0.703
Stage III	37/9	1.84 (0.94-3.31)	0.087		54/57	1.25 (0.76-2.06)	0.382		0/0	NA	NA
Stage IV	5/3	1.08 (0.22-5.44)	0.925		3/13	6.02 (0.52-70.04)	0.003		0/0	NA	NA

Abbreviations: HR, Hazard ratio; 95%CI, 95% confidence interval; AJCC, the American Joint Committee on Cancer.

**Table 3:** The association between eight-lncRNA signature and DFS of NSCLC patients in discovery and validating groups.

Variable	Discovery Group			Validation Group-1				Validation Group-2			
	Number (High/Low)	HR (95%CI)	*P* value		Number (High/Low)	HR (95%CI)	*P* value		Number (High/Low)	HR (95%CI)	*P* value	
**Total**	**228/228**	**2.61 (1.91-3.67)**	**<0.001**		**335/335**	**1.45 (1.11-1.91)**	**0.006**		**65/65**	**3.05 (1.58-5.26)**	**<0.001**	
Gender												
Male	196/160	2.64 (1.74-3.63)	<0.001		209/212	1.31 (0.93-1.86)	0.120		34/26	7.45 (2.16-10.36)	<0.001	
Female	63/107	2.25 (1.23-5.41)	0.013		126/123	1.70 (1.10-2.63)	0.018		31/39	1.28 (0.50-3.25)	0.603	
Smoking history												
Never smoker	2/15	3.89 (0.92-157.30)	0.065		22/29	0.65(0.27-1.60)	0.366		24/43	1.26(0.47-3.42)	0.638	
Ever smoker	20/53	3.26 (1.57-12.45)	0.005		217/237	1.43(1.01-2.02)	0.040		41/22	8.17(1.80-8.60)	0.001	
Current smoker	10/39	1.33 (0.33-5.54)	0.665		84/59	1.62(0.93-2.82)	0.093		0/0	NA	NA	
Histology												
Squamous Carcinoma	107/69	3.08 (1.40-4.67)	0.002		219/196	1.46 (1.00-2.12)	0.048		0/0	NA	NA	
Adenocarcinoma	86/165	2.50 (1.73-5.48)	<0.001		116/139	1.57 (1.05-2.35)	0.021		0/0	NA	NA	
AJCC stage												
**Stage I**	**165/193**	**2.40 (1.61-3.81)**	**< 0.001**		**180/174**	**1.61 (1.06-2.44)**	**0.021**		**43/54**	**2.52 (1.15-5.64)**	**0.022**	
Stage II	46/54	2.20 (1.14-4.66)	0.021		97/86	1.25 (0.76-2.06)	0.382		22/11	2.67 (0.85-5.92)	0.104	
Stage III	35/6	3.68 (1.30-6.70)	0.015		54/57	1.21 (0.65-2.25)	0.552		0/0	NA	NA	
Stage IV	5/4	0.31 (0.00-2.29)	0.221		3/13	NA	0.635		0/0	NA	NA	

Abbreviations: HR, Hazard ratio; 95%CI, 95% confidence interval; AJCC, the American Joint Committee on Cancer.

**Figure 4:**
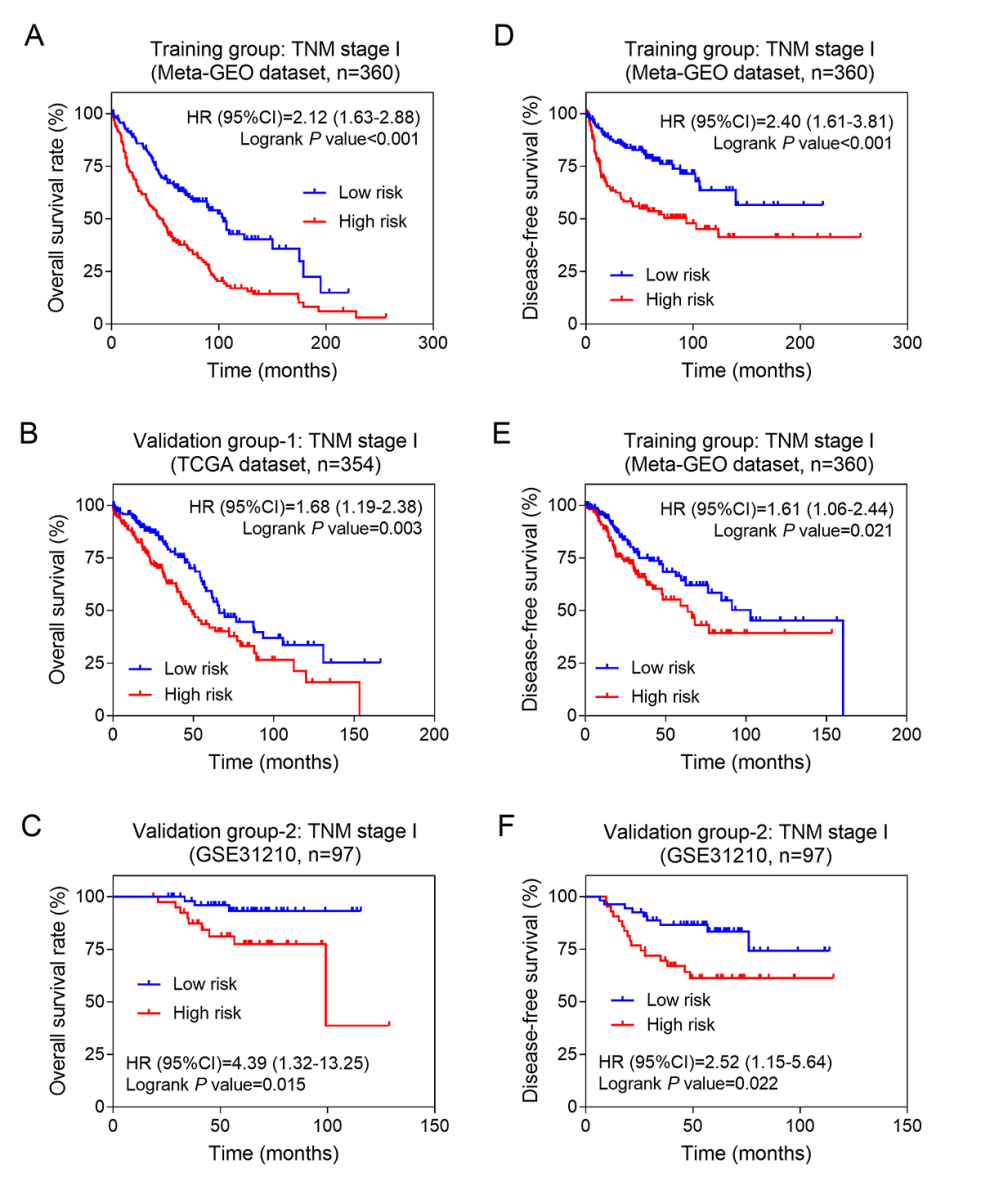
**The eight-lncRNA signature was associated with prognosis in stage I patients.** Kaplan-Meier analysis of the overall survival of patients with stage I in training group (**A**), validation group-1 (**B**) and validation group-2 (**C**); Kaplan-Meier analysis of the disease-free survival of patients with stage I in training group (**D**), validation group-1 (**E**) and validation group-2 (**F**).

Furthermore, we performed stratified analysis in different AJCC stages. The result showed that the eight-lncRNA signature had the ability of predicting prognosis in stage I only. Kaplan–Meier curves for the high- and low-risk groups in stage I patients were plotted. Our data showed that patients with high-risk scores exhibited poorer OS than those with low-risk scores. Above results were confirmed in both the training group (Figure 4A, log-rank test *P* <0.001) and the two validation groups (Figure 4B, log-rank test for validation 1: *P* =0.003; Figure 4C, log-rank test for validation 2: *P* =0.015). Similarly, our results also showed that the eight lncRNAs signature was associated with DFS of NSCLC patients with stage I in three groups (Figure 4D-F). Above findings suggested that the eight lncRNAs signature might be a prognostic biomarker for NSCLC patients with early stage.

### Functional characteristics of eight prognostic lncRNAs

**Figure 5:**
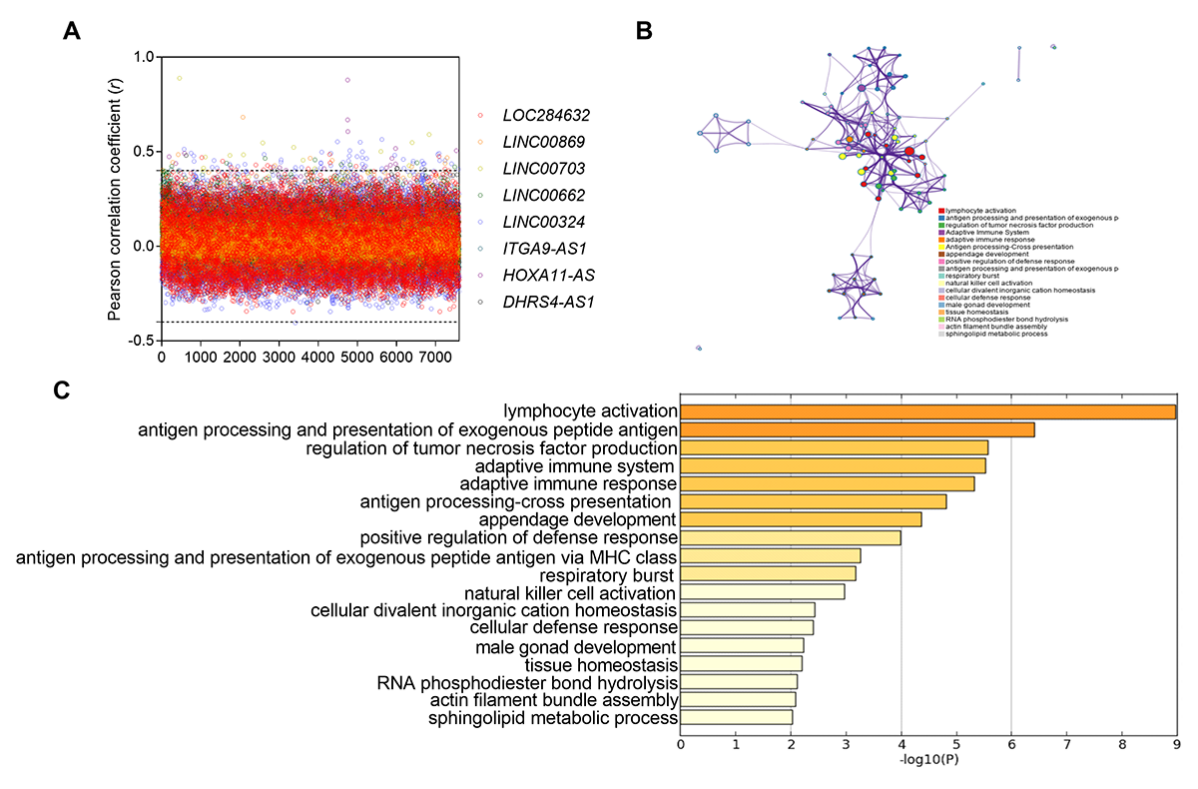
**Functional enrichment results of the co-expressed protein-coding genes with eight lncRNAs.** (**A**) the pearso correlation coefficient between 7600 protein-coding genes and eight lncRNAs in TCGA database. (**B**) Significantly enriched pathways of the 126 correlated genes. (**C**) The functional enrichment map of pathways. Each node represents a GO term. Node size represents the number of gene in the pathways.

To further explore the potential function of the above eight lncRNAs in NSCLC, we analyzed the coexpressed genes with eight lncRNAs by calculating Pearson correlation between the eight-lncRNA signature and 7600 protein-coding genes in TCGA dataset. The screening criteria for the encoded protein genes was that these genes were positively associated with at least one lncRNA (Pearson coefficient > 0.4, *P* < 0.01) (Figure 5A). A total of 126 genes were selected for pathway enrichment analysis. The results showed that the 126 coexpressed genes were mostly enriched in 18 pathways (especially immune regulatory pathways), such as lymphocyte activation, antigen processing and presentation of exogenous peptide antigen, etc (Figure 5B-C). It suggested that these eight lncRNAs might be involved in regulating tumor immune status.

## DISCUSSION

In the present study, we identified a potential eight-lncRNA signature for predicting OS and DFS of elderly NSCLC patients. A total of five GEO and two TCGA datasets were employed in this study. After a comprehensive analysis, eight lncRNA signature was conducted and were identified to be associated with prognosis in elderly NSCLC patients. The ability to predict prognosis has also been confirmed in two other independent datasets. Furthermore, stratified analysis showed that the eight-lncRNA signature had a high predictive accuracy in predicting OS and DFS of NSCLC patients with early stage.

It is well known that population aging has become a global issue. It will cause a rapid increase of primary lung cancer as well as the number of operations for lung cancer among elderly patients. Therefore, effective disease prevention and treatment strategies for the elderly are necessary. During the past few decades, researches on the prevention, diagnosis and treatment of elderly patients with lung cancer have been reported. In a study investigating the efficacy of metronomic vinorelbine in the treatment of patients with advanced unresectable NSCLC, age was found to be an important factor that affected the treatment efficiency [15]. Exploring effective indicators for elderly cancer patients has been drawing increasing attentions. In the present study, we, for the first time, identified a risk model containing eight lncRNAs that can effectively predict the prognosis of elderly patients with NSCLC. Moreover, it can effectively predict overall survival and tumor-free survival at the same time.

Because of the critical limitations on the TNM staging system and other scoring systems today, it is necessary to find new molecular markers to help clinical evaluation of prognosis and diagnosis. A large number of literatures have reported that certain protein-encoding genes and microRNAs can predict the prognosis and diagnosis of lung cancer patients [16,17]. For example, high expression of miR-155 in serum can help diagnose non-small cell lung cancer. The sample of the detection method is convenient to obtain [18]. Moreover, thanks to the development of CHIP technology, a large number of lncRNAs aberrantly expressed in tumor tissues were discovered [19–21]. Many of them have been confirmed to be closely related to the occurrence, development and recurrence of tumors [22,23]. Accumulating evidence suggested that lncRNAs were involved in oncogenic and tumor suppressive pathways indicating a great potential as tumor biomarkers. Furthermore, these dysregulated lncRNAs have already shown great potential as novel molecular biomarkers for diagnosis, prognosis and treatment of cancer. For example, lncRNA *AFAP1-AS1* could affect NSCLC patients’ survival and epigenetically repress *p21* expression which was a key molecular in tumor progression [24]. In our study, instead of looking for a single lncRNA as a predictor of lung cancer prognosis, we found multiple lncRNAs to predict tumor prognosis. In this study, we identified a total of eight lncRNAs (*LOC284632, LINC00869, LINC00703, LINC00662, LINC00324, ITGA9-AS1, HOXA11-AS and DHRS4-AS1*) and built a prognostic formula. Kaplan-Meier analysis results showed this risk score model has good ability in prognosis prediction. Furthermore, we employed two independent group (TCGA and GSE31210 datasets) as validation groups in order to minimize the bias generated by small-scale data analysis. Our results confirmed the eight-lncRNA signature was a robust and reproducible prognostic biomarker.

Stratification analysis based on clinical characteristics was performed in this study. After analyzing the prognostic values in different AJCC stages, we found the eight-lncRNA signature was significantly associated with OS and DFS in patients with stage I. Considering the surgery is the first-line recommend therapy for stage I patients [25], our eight-lncRNA signature could help physicians to predict patients’ prognosis after surgery and to implement effective treatment options. In addition, a large number of studies have been conducted to successfully detect microRNAs in plasma/serum. For example, miR-155 could be sensitively and specifically measured in serum. Overexpression of miR-155 in serum specimens could constitute a diagnostic marker for the early detection of lung adenocarcinoma [18]. Similar as microRNAs, lncRNA plays a huge role in tumor diagnosis and prognosis. Techniques for detecting lncRNA in plasma/serum could contribute to diagnosing disease and predicting prognosis. A study identified plasma HDRF and RDRF which is RNA fragments in plasma/serum derived from lncRNA HOTTIP-005 and lncRNA RP11-567G11.1 in pancreatic cancer (PC). It would to be used as prognostic and diagnostic biomarkers of PC [26]. Therefore, we believe that the expression level and significance of these 8 lncRNAs in the plasma/serum of patients with NSCLC need further study. This would further improve the early diagnosis rate and recurrence rate of patients with NSCLC and improve the survival rate of patients.

Among the eight lncRNAs, five of them, including LOC284632, LINC00869, LINC00703, LINC00662 and HOXA11-AS, acted as protective factors for NSCLC, and the other three lncRNAs (LINC00324, ITGA9-AS1 and DHRS4-AS1) were risk factors. Except for HOXA11-AS and DHRS4-AS1, the other six lncRNAs have not been reported in the literature. Moreover, except for HOXA11-AS, the other 7 lncRNAs in this study were firstly reported as biomarkers in NSCLC. DHRS4-AS1 as a tumor inhibitor functions by preventing the proliferation and invasion, inhibiting the cell cycle progression and promoting the apoptosis of clear cell renal cell carcinoma cells [27]. *HOXA11-AS* has been studied as a oncogene in NSCLC, gastric cancer, liver cancer, osteosarcoma, and breast cancer [28–33]. HOXA11-AS was markedly overexpressed in NSCLC and was associated with patients’ prognosis [28]. Experimental evidences suggested that HOXA11-AS was involved in cellular proliferation, migration and invasion. HOXA11-AS also mediated cisplatin resistance of NSCLC cells [34]. Several signaling pathways, such as TGF-beta (TGF-β) pathway, was regulated by HOXA11-AS [35]. This provides new ideas for the study of non-small cell carcinoma machines.

Due to the unclear function of 8 lncRNAs in NSCLC, we also performed pathway enrichment analysis to find the potential biological functions of eight lncRNAs. The mostly enriched pathways were involved in immune regulation, including lymphocyte activation and antigen processing, presentation of exogenous peptide antigen and regulation of tumor necrosis factor (TNF) production, etc. It indicated that the eight-lncRNA might function as tumor immunomodulatory in NSCLC. Nowadays, the investigations of lncRNA in tumors mainly focused on gene imprinting and tumor cell differentiation. A few literatures also reported that lncRNAs were involved in regulating immune response of cancer patients. It was reported that CD8+ T cells and CD4+ T cells expressed a large number of lncRNA genes, many of which were specific to lymphocytes and were dynamically regulated during differentiation or activation [36,37]. Moreover, we also predicted that the eight-lncRNA might affect the production of tumor necrosis factor (TNF). Our above findings need further experimental studies to confirm.

In summary, we identified an eight-lncRNA signature to predict NSCLC patients’ OS and DFS. The eight-lncRNA signature showed great potential of prognostic prediction of patients, particularly in those with early stage. To our knowledge, this was the first study to identify lncRNA signature in elderly NSCLC patients. Our findings provided evidence of developing effective prognostic biomarkers for NSCLC patients.

## MATERIALS AND METHODS

### Patient information and study design

A total of seven datasets which contain genetic information and clinical data of NSCLC patients were selected in the study. Five of them (GSE30219, GSE31546, GSE37745, GSE50081 and GSE31210) were downloaded from the Gene Expression Omnibus (GEO) and two (TCGA-LUSC and TCGA-LUAD) from The Cancer Genome Atlas (TCGA) websites. Among them, four GEO datasets (GSE30219, GSE31546, GSE37745 and GSE50081) were integrated as a training group via data normalization, including 456 patients. Meanwhile, 670 patients from TCGA dataset (combination of TCGA-LUSC and TCGA-LUAD) and 130 patients from another GEO dataset (GSE31210) were employed as two independent validation groups. The patients included in this study were all NSCLC patients with >=60 years old. Patients under the age of 60 and patients with missing or no clinical data were excluded. The clinicopathological parameters of the HCC patients in each group are listed in Table 4.

**Table 4:** Clinical features of elderly patients (age>=60 years) with NSCLC in the training and validating groups.

Features	Training group (n=456)	Validation group-1 (n=670)	Validation group-2 (n=130)
Age, no (%)			
<75 years	353	486	123
>=75 years	103	184	7
Gender, no (%)			
Male	313 (68.6)	421 (62.8)	60 (46.2)
Female	143 (31.4)	249 (37.2)	70 (53.8)
Smoking status, no (%)			
Never smoker	17 (12.5)	51 (7.9)	67 (51.5)
Ever smoker	71 (52.2)	454 (70.1)	63 (48.5)
Current smoker	48 (35.3)	143 (22.0)	
Pathological grade, no (%)			
Squamous Carcinoma	236 (39.9)	415 (61.9)	NA
Adenocarcinoma	220 (60.1)	255 (38.1)	NA
AJCC stage, no (%)			
I	313 (68.4)	356 (53.3)	97 (74.6)
II	89 (19.6)	185 (27.6)	33 (25.4)
III	46 (10.2)	112 (16.7)	NA
IV	8 (1.8)	17 (2.4)	NA

Abbreviations: AJCC, the American Joint Committee on Cancer.

### Normalization and lncRNA annotation of GEO data

Because of the inconsistency of gene profiling for four GEO datasets (GSE30219, GSE31546, GSE37745 and GSE50081), the quantile normalization using Robust Multi-Array Average (RMA) method were performed in the raw data which were downloaded as probe-level CEL files. The Affymetrix U133 Plus 2.0 which downloaded from Affymetrix website (http://www.affymetrix.com) contained 2986 lncRNA-specific probes.

### Construction of the risk formula for prognostic prediction

Firstly, the lncRNAs whose expression levels cannot be detected (value=0) in more than 10% of all samples were eliminated. Then Univariate Cox proportional hazards regression was performed for the lncRNAs that were significantly associated with the OS of elderly patients with NSCLC in the training group. The lncRNA with a *P* value of less than 0.05 was included in the subsequent analysis. Next, stepwise and multivariate Cox regression model was used to identify optimal lncRNAs which is independently associated with prognosis. Finally, a prognostic risk formula was established based on a linear combination of the expression level of these lncRNAs multiplied by the regression coefficients derived from the multivariate Cox regression model as mentioned above.

### Statistical analysis

Cox proportional hazards regression was used to identify survival-related biomarkers. Comparison of prognosis between high-risk group and low-risk group was performed by Kaplan-Meier survival curves and log-rank test. Time dependent ROC curve was plotted to assess the specificity and sensitivity of the prognostic prediction. The above analyses were performed using R (version 3.3.1). The stratification analysis based on clinicopathological parameters and univariate and multivariate Cox regression analyses were performed using SPSS software (version 24.0).
